# A New Reference Genome Shows the One-Speed Genome Structure of the Barley Pathogen *Ramularia collo-cygni*

**DOI:** 10.1093/gbe/evy240

**Published:** 2018-10-29

**Authors:** Remco Stam, Martin Münsterkötter, Saurabh Dilip Pophaly, Like Fokkens, Hind Sghyer, Ulrich Güldener, Ralph Hückelhoven, Michael Hess

**Affiliations:** 1 Chair of Phytopathology, School of Life Sciences Weihenstephan, Technische University Munich, Germany; 2 Functional Genomics and Bioinformatics, Research Centre for Forestry and Wood Industry, University of Sopron, Hungary; 3 Institute of Bioinformatics and Systems Biology, Helmholtz Centre Munich, Germany; 4 Section of Population Genetics, School of Life Sciences Weihenstephan, Technische Universität München, Germany; 5 Molecular Plant Pathology, Swammerdam Institute for Life Sciences, University of Amsterdam, The Netherlands; 6 Department of Bioinformatics, School of Life Sciences Weihenstephan, Technische University Munich, Germany; 7 Department of Evolutionary Biology, Evolutionary Biology Centre, Uppsala University, Sweden and Division of Evolutionary Biology, Faculty of Biology II, Ludwig-Maximilians-Universität München, Germany

**Keywords:** fungi, phytopathology, genome, dothideomycetes, barley pathogen

## Abstract

Ramularia leaf spot has recently emerged as a major threat to barley production world-wide, causing 25% yield loss in many barley growing regions. Here, we provide a new reference genome of the causal agent, the Dothideomycete *Ramularia collo-cygni.* The assembly of 32 Mb consists of 78 scaffolds. We used RNA-seq to identify 11,622 genes of which 1,303 and 282 are coding for predicted secreted proteins and putative effectors respectively.

The pathogen separated from its nearest sequenced relative, *Zymoseptoria tritici* ∼27 Ma. We calculated the divergence of the two species on protein level and see remarkably high synonymous and nonsynonymous divergence. Unlike in many other plant pathogens, the comparisons of transposable elements and gene distributions, show a very homogeneous genome for *R. collo-cygni*. We see no evidence for higher selective pressure on putative effectors or other secreted proteins and repetitive sequences are spread evenly across the scaffolds. These findings could be associated to the predominantly endophytic life-style of the pathogen. We hypothesize that *R. collo-cygni* only recently became pathogenic and that therefore its genome does not yet show the typical pathogen characteristics. Because of its high scaffold length and improved CDS annotations, our new reference sequence provides a valuable resource for the community for future comparative genomics and population genetics studies.

## Introduction

The filamentous ascomycete fungus *Ramularia collo-cygni* was first described in 1893 as *Ophiocladium hordei* (Cavara 1893). It is the biotic agent of ramularia leaf spot (RLS) (Oxley and Havis 2004), a disease typically occurring late in the growing season on the upper canopy (Salamati and Reitan 2006). Since the mid-1980s it has become the major pathogen in many barley growing regions worldwide and quickly developed resistance to major fungicides (Matusinsky et al. 2011; Havis et al. 2015; Piotrowska et al. 2017). It can now be detected in barley samples worldwide (Havis et al. 2015) and in infected fields it is estimated to cause losses around 25% of the yield potential through a significant decrease of kernel size and quality (Harvey 2002).

A draft genome assembly of *R. collo-cygni* strain DK05, isolated in Denmark, had been published previously (McGrann et al. 2016). Like its closest sequenced relative *Zymoseptoria tritici*, *R. collo-cygni* has few plant cell wall degrading enzyme genes and a large number of gene clusters associated with secondary metabolite production. These findings are thought to be linked to the relatively long period of asymptomatic growth of both pathogens inside the host.

We present an independent draft genome of a strain isolated in southern Uruguay. The significantly increased scaffold size enabled several analyses related to genome architecture. Moreover, our improved annotation allows for more reliable identification of genes that are under positive selection and may be involved in pathogenicity.

## Materials and Methods

### Genome Assembly and Annotation

A detailed description of the genome sequencing strategy, assembly and annotation can be found in the supplementary files. In short: sequencing was done by Eurofins Genomics GmbH, Germany, using a short distance library (SD) (insert size, 500 bp, paired-end sequencing 2 × 150 bp) and a LJD (jumping distance 8-kb, paired-end sequencing 2 × 300 bp). RNA-seq was performed using TruSeq Rapid PE Cluster (PE-402-4001) and the TruSeq Rapid SBS (FC-402-4001) Kits.

The assembly was performed by ALLPATHS-LG (Gnerre et al. 2011) using ∼100-fold SD and ∼30-fold LJD coverage. Gene models were generated by 1) Fgenesh (Salamov and Solovyev 2000), 2) GeneMark-ES (Ter-Hovhannisyan et al. 2008), and 3) AUGUSTUS (Stanke et al. 2006). RNA-seq transcripts were assembled using Trinity (Grabherr et al. 2011). Gene models were visualized in Gbrowse (Donlin 2009), allowing manual validation of coding sequences. The best fitting model per locus was selected manually and gene structures were adjusted by splitting or fusing of gene models or redefining exon–intron boundaries if necessary.

The protein coding genes were analyzed and functionally annotated using the PEDANT system (Walter et al. 2009). We combined SecretomeP1.1 (Bendtsen, Jensen, et al. 2004), SignalP3 (Bendtsen, Nielsen, et al. 2004), and SignalP4.0 (Petersen et al. 2011) with TargetP1.1 (Emanuelsson et al. 2007), TMHMM2.0 (Krogh et al. 2001) to predict secreted proteins. For effector prediction, this set was submitted to EffectorP 2.0 (Sperschneider et al. 2016). Repetitive elements were identified using the RepeatModeler pipeline (smith 2013), which is based on RepeatScout (Price et al. 2005).

### Further Analyses

Orthologous genes for 10 main housekeeping genes we selected from the proteomes of *Urug2*, 15 closely related species and three more distal species that are available from the FunyBASE (Marthey et al. 2008). We performed a ClustalW pairwise alignment and multiple alignment with standard parameters for the combined proteins of all species (*An*, *Aspergillus nidulans*; *Bc*, *Botrytis cinerea*; *Bg*, *B.graminis*; *Cb*, *Cercospora beticola*; *Cg*, *Colletotrichum graminicola*; *Ds*, *Dothistroma septosporum*; *Fg*, *Fusarium graminearum*; *Ff*, *Fusarium fujikuroi*; *Mf*, *Pseudocercospora fijiensis*; *Mo*, *Magnaporthe grisea*; *Pi*, *Piriformospora indica*; *Pr*, *P.tritici-repentis*; *Pt*, *P.teres f. teres*; *Rc*, *R.collo-cygni*; *Sn*, *P.nodorum*; *Sm*, *Sphaerulina musiva*; *Um*, *Ustalago maydis*; *Zt*, *Z.tritici*; *Outgroup*, *Laccaria bicolor*) using Mega X (Kumar et al. 2018) Dating of the Zt–Rc split was done using the RelTime method (Tamura et al. 2012), where Lb was used as an outgroup and the data were calibrated using the Um to An split (Taylor and Berbee 2006).

Orthologous genes to all single copy genes were identified in the Zt proteome (BLASTP *e*-value: 10^−10)^, only reciprocal matches were used and globally aligned using t-coffee (default parameters) (supplementary table S5, Supplementary Material online). Amino acids were replaced by codons from the CDS sequence using pal2nal (Suyama et al. 2006). The d*N*/d*S* ratios were calculated with PAML (Yn00 command) (Yang and Nielsen 2000). Intergenic distances and TE distances were calculated using bedtools (closest) (Quinlan and Hall 2010). Genome alignments between the assembly of Urug2 and that of DK05 were inferred using nucmer (with –maxmatch, otherwise default options) from the MUMmer package (version 3.23) and alignments that span >1 kb and are >90% identical in sequence were plotted using Gnuplot.

## Results

### Genome Properties

Combining short distance with long jumping distance libraries (LJDs), we obtained a ∼32-Mb assembly of *R. collo-cygni* isolate Urug2, in 78 scaffolds with an N50 scaffold size of 2.1 Mb, compared with 576 scaffolds and an N50 of 0.21 in the previous DK05 assembly (table 1). Dot plot analysis comparing both Urug2 to DK05, shows strong linearity suggesting little to no overassembly (supplementary fig. S1, Supplementary Material online). To bolster gene annotations, we sequenced mRNA isolated from *R. collo-cygni* grown under six axenic conditions to generate a diverse set of transcripts. Using these data mapped to the genome, the annotation was manually corrected gene by gene, yielding a more reliable set of gene models (example shown in supplementary fig. S2, Supplementary Material online). The curated *R. collo-cygni* genome contains 11,622 protein coding genes of which 11,125 show expression evidence (95.7%, supplementary table S1, Supplementary Material online). We predicted 1,303 secreted proteins ranging in length from 41 to 3,256 amino-acids, representing around 9% of the predicted proteome, among which 282 effector candidates genes (putative effectors) (2%) (supplementary table S2, Supplementary Material online). These numbers are an increase over the previous *R. collo-cygni* assembly that contained 1,053 putative secreted proteins and 150 effector candidates.

**Table 1:** Summary Statistics for Our Urug2 Genome, as well as Other Related Fungi

* *	*Ramularia collo-cygni*	*Ramularia collo-cygni*	*Phaeosphaeria nodorum*	*Pyrenophora tritici-repentis*	*Pyrenophora teres-teres*	*Zymoseptoria tritici*
Isolate	Urug2	DK05 Rcc001	SN15	BRP-ToxAC	0-1	IPO323
Genome size (Mb)	32.3	30.3	37.2	37.8	46.5	39.7
Chromosomes	n.d.	n.d.	n.d.	n.d.	n.d.	21
Scaffolds	78	576	107	47	86	21
N50 scaffold (Mb)	2.1	0.21	0.17	1.9	1.73	Finished
GC-content (%)	49.7	51.4	50.2	51	46	51.7
Protein coding genes	11,622	11,617	14,885	12,141	11,541	10,933
Gene density (number of genes per Mb)	383	384	402	321	248	276
Total secreted Protein	1,303	1,053^a^	2,172	1,433	1,357	1,252
Number of predicted effectors^a^	282	150^a^	625	382	337	380

Note.—Genome statistics were taken from the respective publications for each organism. The number of putative secreted proteins and effector candidates was recalculated for each species except DK05, using our newest pipeline.

a DK05 gene/protein models were not publicly available.

The general genome features are comparable to other Dothideomycete plant pathogens with available genome sequences (*Parastagonospora nodorum* SN15, Hane et al. 2007; Syme et al. 2013, *Pyrenophora tritici-repentis* BFP-ToxAC, Manning et al. 2013, *Pyrenophora teres f. teres* 0-1, Wyatt et al. 2018, and *Z.tritici* IPO323, Goodwin et al. 2011; table 1). We estimated genome completeness using BUSCO (version 3.0.1). 96.8% of the genes were detected as complete and single-copy in the *R. collo-cygni* Urug2 assembly. This exceeds the completeness of *R. collo-cygni DK05* (93.9%) and *Z. tritici IPO323* (92.5%) and is similar to *P.teres f.teres* 0-1 (supplementary table S3, Supplementary Material online).

### Gene Expression Analysis

We saw similar levels of gene expression in all six tested media, with a slightly larger fraction of genes expressed in Barley Straw Agar (BSA), a host-mimicking agar medium compared with neutral or pH-adjusted media (supplementary table S1, fig. S3*A*, Supplementary Material online). We found a larger number of differentially expressed genes (DEGs) (>4-fold change) in BSA (supplementary fig. S3*B*, Supplementary Material online). We expected that gene expression in BSA most closely resembles infection of the plant. Indeed, we found that the fraction of putative secreted proteins and effector candidates is two times higher in the BSA differentially expressed genes than in the genome as a whole (resp. 22% and 5%).

### Strong Divergence from *Z. tritici*

**Fig. 1.**
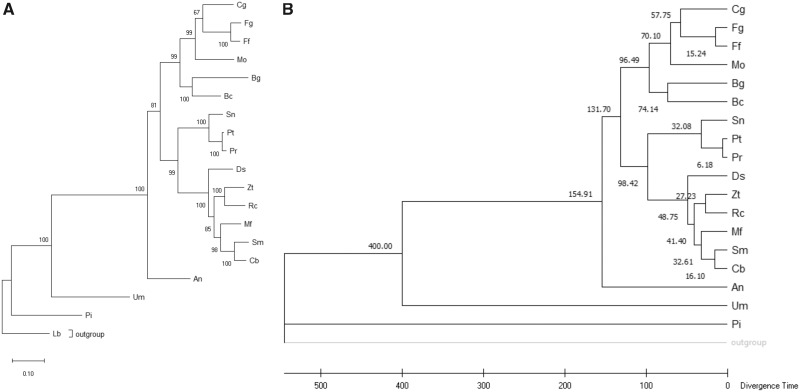
—Phylogenetic tree showing the relationship between *Ramularia collo-cygni* and 16 other fungi. (*A*) Maximum likelihood phylogenetic tree based on the analysis of 10 housekeeping genes. Reported on the nodes are bootstrap values for 1,000 bootstraps. (*B*) Timetree results. Reported on the nodes are the divergence times in mya. *Bc*, *Botrytis cinerea*; *Bg*, *Blumeria graminis*; *Cg*, *Colletotrichum graminicola*; *Cb*, *Cercospora beticola*; *Ds*, *Dothistroma septosporum*; *Fg*, *Fusarium graminearum*; *Ff*, *Fusarium fujikuroi*; *Mf*, *Pseudocercospora fijiensis*; *Mo*, *Magnaporthe grisea*; *Pr*, *Pyrenophora tritici-repentis*; *Pt*, *Pyrenophora teres f. teres*; *Rc*, *R. collo-cygni*; *Sn*, *Parastagonospora nodorum*; *Sm*, *Sphaerulina musiva*; *Zt*, *Zymoseptoria tritici*; *An*, *Aspergillus nidulans*; *Um*, *Ustalago maydis*; *Pi*, *Piriformospora indica*; *Outgroup, Laccaria bicolor*.

**Fig. 2.**
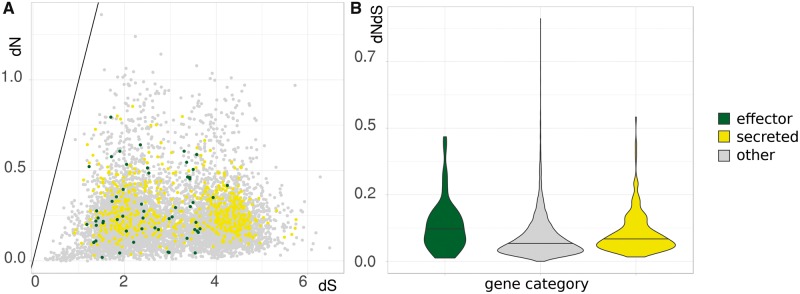
—d*N*/d*S* between *Ramularia collo-cygni* and *Zymoseptoria tritici*. (*A*) Scatter plot of the dS (*x* axis) against dN (*y* axis) for each predicted protein in a pairwise comparison between *R. collo-cygni* and *Z. tritici.* (*B*) Violin diagrams of the d*N*/d*S* ratio for predicted proteins in a pairwise comparison between *R. collo-cygni* and *Z. tritici.* Data colored based on whether the proteins are predicted to be putatively secreted proteins, putative effectors or other, nonsecreted, proteins. Horizontal bar depicts the median.

**Fig. 3.**
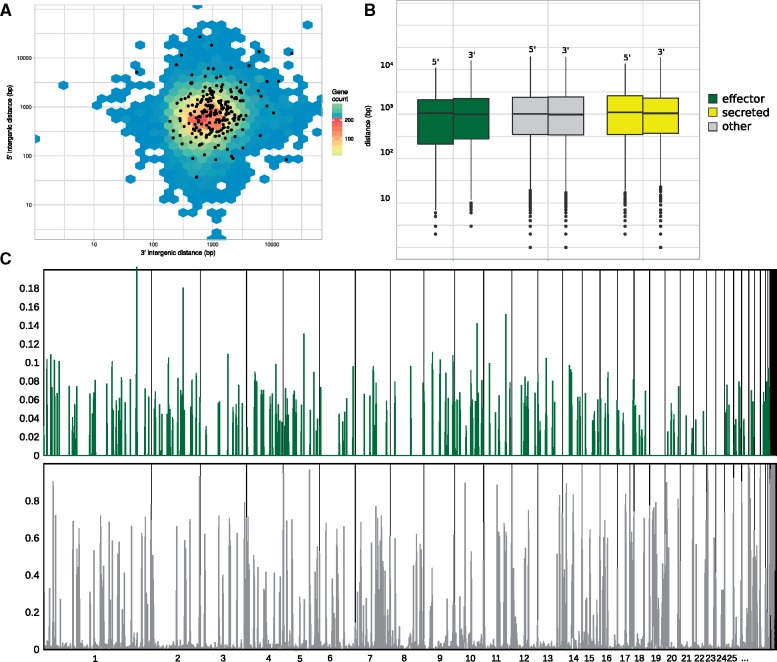
—Intergenic distance and TEs in *R. collo-cygni*. (*A*) Density plot of the intergenic distances on the 5′ end (*x* axis) against the 3′ end (*y* axis) for each predicted gene on the genome. Distances for all genes are binned in hexagons and colored using a blue to red scale, red indicating the largest amount of genes per hexagon*.* Putative effectors are plotted as separate black dots. (*B*) Box plots of the distance to the nearest TE for each gene coding for predicted nonsecreted (grey), putative secreted (yellow), or effector (green) proteins. (*C*) Density of putative effector encoding genes (top, green) and transposable elements (bottom, grey) plotted in 10-kb nonoverlapping sliding windows along the genome (*x* axis). Density is defined as number of basepairs that is part of a putative effector or TE in the window (*y* axis). A close-up of scaffolds 21-35 is provided in supplementary Fig. S4.

We reconstructed a phylogenetic tree of *R. collo-cygni*, with 15 more closely and three more distantly related species (fig. 1) and confirm that *R. collo-cygni* falls within the Mycosphaerellaceae clade of the Dothideomycete class. *Ramularia collo-cygni* (Rc) diverged from the closest sequenced relative, *Z. tritici* (Zt) 27 Ma. To gain insight in the differentiation of *R. collo-cygni* from *Z. tritici*, we calculated the ratio of non-synonymous over synonymous substitutions (d*N*/d*S*) (fig. 2). The very high d*S* indicate that these two species have significantly diverged since the split. When comparing the d*N*/d*S* between the two species for the putative secreted proteins and putative effectors, we find that the putative secreted proteins show a slightly higher d*N*/d*S* ratio than nonsecreted proteins (Dunn’s multiple comparisons Kruskal–Wallis test, *P* = 4.7 × 10^−16^). For the effectors this difference is not significant (*P* = 0.16). Effectors and secreted proteins also show no significant differences (*P* = 0.2). In terms of absolute values and outliers, there are no putative effectors that stand out. Similar results can be observed when comparing differentially expressed genes on BSA. As mentioned above, these genes are hypothesized to be important for virulence on barley, yet there are no significant differences in d*N*/d*S* between these BSA up- or down regulated genes and nonDEGs or between up or down regulated genes in general (fig. 3*C*, Dunn’s multiple comparisons Kruskal–Wallis test, *P* > 0.01).

### The *R. collo-cycni* Genome Is Relatively Repeat Poor and Homogeneous

We compared the content of noncoding sequences and repeat sequences like DNA transposons and other transposable elements (TEs) (supplementary table S4, Supplementary Material online). In terms of repeat sequence content, *R. collo-cygni* is placed at the low end of the spectrum amongst Dothideomycetes. Only 6% of the genome consists of TEs, whereas in *P. tritici-repentis*, *P. teres f. teres*, and *Z. tritici* this is 21%, 38%, and 17%, respectively. Next, we compared the distance of predicted genes to its nearest repeat sequence as well as the general intergenic distance. Close association of genes to TEs and large intergenic distance for regions with high effector content are features of a so-called “two-speed-genome” and often associated with accelerated evolution (Raffaele and Kamoun 2012). Figure 3*A* shows that intergenic distances are not differently distributed between putative effectors genes or other genes and the mean values for the difference to the nearest TE for putative effectors and putative secreted proteins are not significantly different from the distances for not secreted proteins (effector: 3′: 1,739 bp, 5′: 1,646 bp, secreted 3′: 1,659 bp, 5′: 1,715 bp, nonsecreted: 3′: 1724, 5′: 1727) (Dunn’s multiple comparisons Kruskal–Wallis test, *P* > 0.1). Lastly, we also do not find a significant correlation between the number of TEs per kb/scaffold or the number of effectors or secreted proteins (Spearman rho, *P* > 0.01, fig. 3*C*; supplemantary fig. S4).

## Discussion

A first draft genome of *R. collo-cygni* (isolate DK05) had been available since 2016. The data suggested a genetic composition that might at least partially explain the lifestyle of *R. collo-cygni*, which is characterized by a long endophytic phase throughout the life cycle of the host and an intense parasitic phase during crop senescence (McGrann et al. 2016). We generated an independent draft genome and annotation for another isolate (Urug2) to get better insights in the *R. collo-cygni* genome structure. We assembled the 32-Mb genome into only 78 scaffolds with *11*,*622* high confidence genes. Our expression data greatly helped with gene annotations and provide interesting insights in genes expressed under different axenic conditions. This will help researchers to verify target gene candidates for functional studies, yet to truly understand gene expression during the infection process, additional RNA-Seq from infected plant tissue will be required.

Our sequencing approach allowed for comparative studies and confirmed that unlike many other pathogens *R. collo-cygni* did not undergo any genome expansions since it diverged from its nearest sequenced sister species 27 Ma. Unlike what can be seen between certain fungal and oomycete species, where the numbers of genes in some effector families differ up to an order of magnitude (Stam et al. 2013) the numbers of putative effectors in *R. collo-cygni* are comparable with that of related fungi.

We performed pairwise comparisons of the coding sequences of *R. collo-cygni* and the related wheat pathogen *Z. tritici*. The d*N*/d*S* ratio has a simple and intuitive interpretation of selection pressure, but comes with limitations, especially when dS is high (Kryazhimskiy and Plotkin 2008). However, our analyses provide interesting insights. Contrary to the phenomenon observed in a large number of other plant pathogens, we see little evidence for accelerated evolution of secreted proteins, putative effectors or genes that are likely differentially expressed during infection, between *R. collo-cygni* and *Z. tritici*. This is in stark contrast to for example *Colletotrichum species*, where high d*N*/d*S* of effectors was associated with the switch from endophytic to parasitic lifestyle (Hacquard et al. 2016). This however, leaves the possibility that this switch is still ongoing *in R. collo-cygni.* The species can also infect other graminaceous hosts, but with less severe symptoms it often appears endophytic (Kaczmarek et al. 2017).

From *Z. tritici*, *R. collo-cygni’s* nearest sequenced relative, we know that rapid pathogen evolution can often be associated with high repeat content of the genome (Poppe et al. 2015), or close physical association of TEs with putative effector genes, which results in a so-called “two-speed” genome architecture. Also for other Dothideomycetes like *P.nodorum* (Richards et al. 2018) and *P. teres f. teres* (Wyatt et al. 2018) this two speed genome is evident. In *P. tritici-repentis* TE content has even be directly associated with the pathogenicity of the strains (Manning et al. 2013). In *R. collo-cygni* repeat content is low and secreted proteins or putative effectors are not closely associated with TEs. Other examples of typical “one-speed-genome” pathogen is the biotrophic barley pathogen *Blumeria graminis* (Frantzeskakis et al. 2018). However, that species is relatively unrelated and has a very different lifestyle. Comparing the mechanisms that drive evolution of pathogenicity in these two diverse one-speed-genome barley pathogens will be particularly interesting. Also, additional investigation is TEs and the relatedness to host, host specificity and aggressiveness as a pathogen in *R. collo-cygni* and other Dothideomycetes will likely teach us more on how this diverse class of cereal pathogens arose and became successful. Our new reference genome and improved annotation provides a starting point for doing so.

## Supplementary Material

Supplementary data are available at *Genome Biology and Evolution* online.

## Supplementary Material

Supplementary Data
