# Supplementary material for: A New Reference Genome Shows the One-Speed Genome Structure of the Barley Pathogen Ramularia collo-cygni
Source: Genome Biol Evol. 2018 Oct 29;10(12):3243–9. doi: 10.1093/gbe/evy240 (PMC6301796; doi:10.1093/gbe/evy240)
Supplement: Supplementary Data [file evy240_supp.zip › Figure S3.pdf]

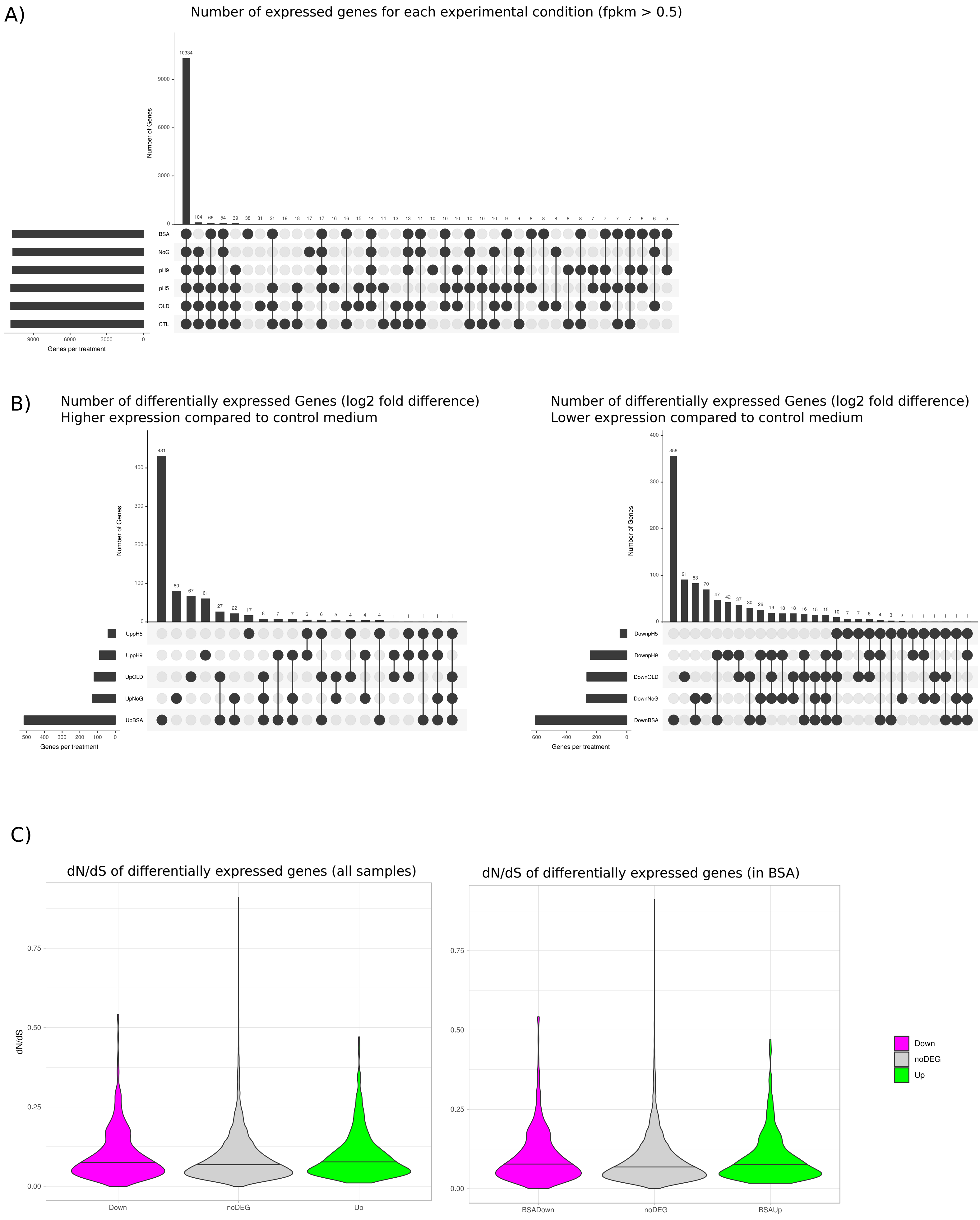

A)
Number of expressed genes for each experimental condition (fpkm > 0.5)
Number of Genes
Genes per treatment
BSA
NoG
pH9
pH5
OLD
CTL
B)
Number of differentially expressed Genes (log2 fold difference)
Higher expression compared to control medium
Number of differentially expressed Genes (log2 fold difference)
Lower expression compared to control medium
Number of Genes
Genes per treatment
UppH5
UppH9
UpOLD
UpNoG
UpBSA
DownpH5
DownpH9
DownOLD
DownNoG
DownBSA
C)
dN/dS of differentially expressed genes (all samples)
dN/dS of differentially expressed genes (in BSA)
dN/dS
Down
noDEG
Up
BSADown
BSAUp
