# Supplementary material for: A New Reference Genome Shows the One-Speed Genome Structure of the Barley Pathogen Ramularia collo-cygni
Source: Genome Biol Evol. 2018 Oct 29;10(12):3243–9. doi: 10.1093/gbe/evy240 (PMC6301796; doi:10.1093/gbe/evy240)
Supplement: Supplementary Data [file evy240_supp.zip › Legends supplementary materials.docx]

**Legends supplementary materials**

**Figure S1:** Dot plot representation of the comparison of the previously published *R. collo-cygni* DK05 genome and our Urug2 genome. Only alignments that span more than 1 kb in both the query and the reference, and are more than 90% identical are included. Dots are coloured according to % identity as indicated in the colour bar to the right.

**Figure S2:** Genome browser screenshot showing the alternative gene models called using gene predictors and the accuracy that the RNA-seq brings in choosing the right model. The gene in the red box matches the RNA-Seq data, the alternative call is discarded.

**Figure S3:** A) The number of genes with expression evidence (fkpm > 1) under different axenic conditions. B) The number of genes that show higher or lower expression values (log2-fold change) under different axenic conditions when compared with standard growth medium. C) Differences in dN/dS between genes that have higher (magenta) and lower (green) expression values on BSA or no changes (grey) compared to other axenic media.

**Figure S4** Density of putative effector encoding genes (green) and transposable elements (grey) for scaffolds 21-34 plotted in 10 kb non-overlapping sliding windows along the genome (x axis). Density is defined as number of basepairs that is part of a putative effector or TE in the window (y axis). Generally very few effectors are found on these scaffolds and association with TEs is not evident.

**Table S1:** Summary of the RNA-seq data. FPKM values are reported for each gene in different media and can be used to calculate differential expression.

**Table S2:** Gene identifiers of all predicted putative secreted proteins and putative effectors

**Table S3**: Summary statistics for BUSCO output.

**Table S4**: Comparison of transposable and repeat elements in *R. collo-cygni* and related species.

**Table S5**: *R. collo-cygni* and *Z. tritici* orthologs and dN/dS calculations
