# Supplementary figures and images for: A New Reference Genome Shows the One-Speed Genome Structure of the Barley Pathogen Ramularia collo-cygni
Source: Genome Biol Evol. 2018 Oct 29;10(12):3243–9. doi: 10.1093/gbe/evy240 (PMC6301796; doi:10.1093/gbe/evy240)

Ramularia collo-cygni DK05

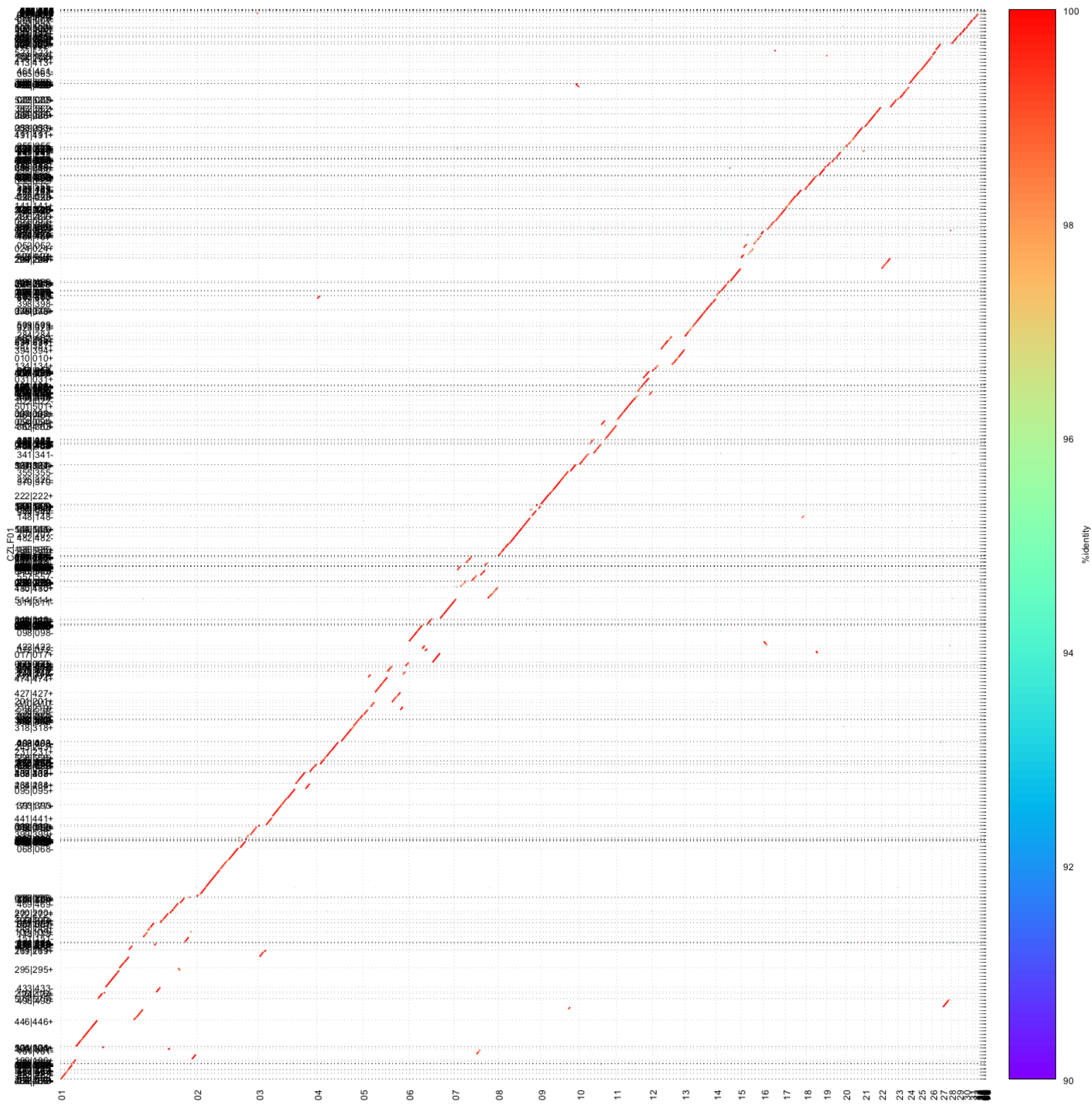

Supplement: Supplementary Data [file evy240_supp.zip › Figure S1.pdf]

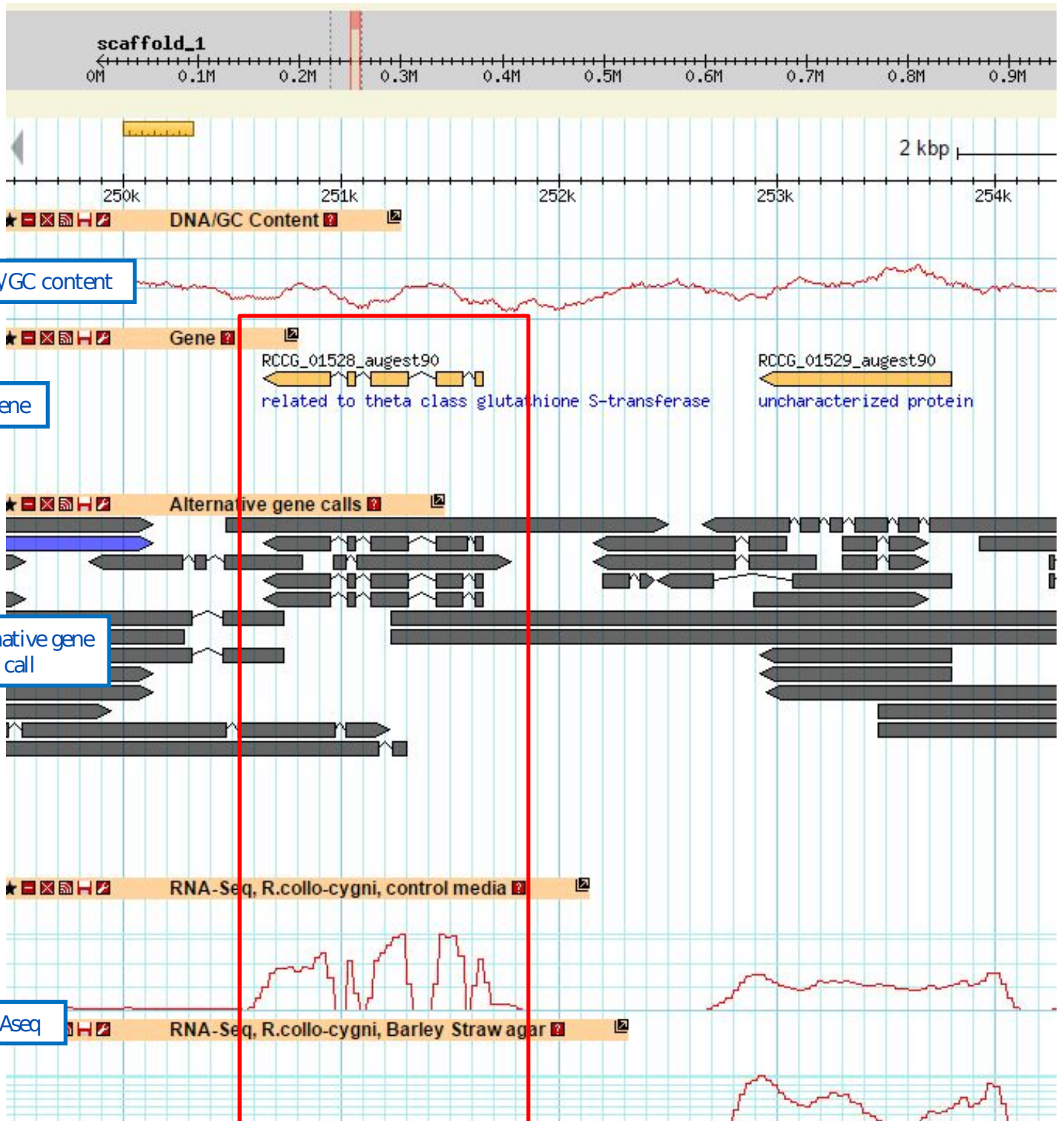

Supplement: Supplementary Data [file evy240_supp.zip › Figure S2.pdf]

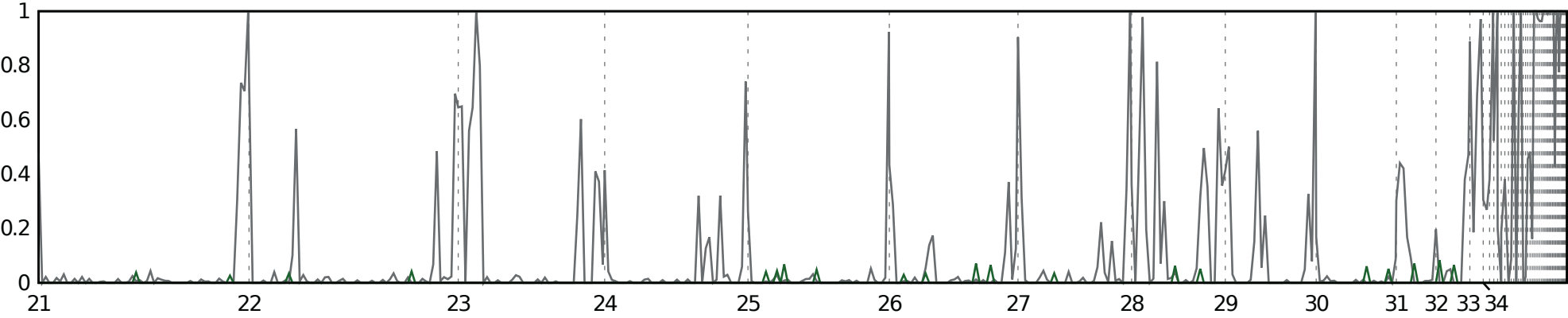

Supplement: Supplementary Data [file evy240_supp.zip › Figure S4.pdf]
