# Supplementary material for: A New Reference Genome Shows the One-Speed Genome Structure of the Barley Pathogen Ramularia collo-cygni
Source: Genome Biol Evol. 2018 Oct 29;10(12):3243–9. doi: 10.1093/gbe/evy240 (PMC6301796; doi:10.1093/gbe/evy240)
Supplement: Supplementary Data [file evy240_supp.zip › Stametal_SuppData.tar.gz!/Table S3 Busco.docx]

**S3 Table. BUSCO single-copy analysis, performed in gene set (protein) assessment mode on the library pezizomycotina_odb9.**

| **Species** | **Strain** | **BUSCO notation assessment results** |
| --- | --- | --- |
| *Ramularia collo-cygni* | URUG2 | C:97.4%, F:1.8%, M:0.8%, n:3156 |
| *Ramularia collo-cygni* | DK05 | C:94.0%, F:1.8%, M:4.2%, n:3156 |
| *Pyrenophora teres-teres* | 0-1 | C:97.7%, F: 1.2%, M:1.5%, n:1315* |
| *Zymoseptoria tritici* | IPO323 | C:92.6%, F:5.2%, M:2.2l%, n:3156 |

C: Complete; S: Single-copy; D: Duplicated; F: Fragmented; M: Missing

* Data taken from Wyatt et al. 2018
