# Supplementary material for: A New Reference Genome Shows the One-Speed Genome Structure of the Barley Pathogen Ramularia collo-cygni
Source: Genome Biol Evol. 2018 Oct 29;10(12):3243–9. doi: 10.1093/gbe/evy240 (PMC6301796; doi:10.1093/gbe/evy240)
Supplement: Supplementary Data [file evy240_supp.zip › Stametal_SuppData.tar.gz!/Extended_Methods_final.pdf]

Method Supplements for Stam et al. 2018

A new reference genome shows the one-speed genome structure of the barley pathogen Ramularia collo-cygni

Fungal isolates and culture maintenance

The isolate Urug2 (isolated in our laboratory from barley leaves collected in southern Uruguay, close to Colonia (-34.334326, -57.691932)) was used for the genome sequencing and expression analysis. It was stored in screw-cap test tubes that contained sterilized one fourth strength potato dextrose broth (¼ PDB) (Carl Roth GmbH + Co. KG). Mycelium of the isolate was transferred to one fourth strength potato dextrose agar (¼ PDA) (Carl Roth GmbH + Co. KG) that was contained in 90-mm-diameter plastic petri dishes.

Cultures media and incubation conditions

R. collo-cygni cultures were maintained 9-cm petri dishes incubated at room temperature in the dark. For the genome sequencing, the isolate was grown on alkylester medium (AE) (10 g yeast extract, 0.5 g MgSO4 *7 H2O, 6 g NaNO3, 0.5 g KCl, 1.5 g KH2PO4, 20 g Bacto Agar, 20 mL glycerol for 1000 ml of final volume) for 7 days.

Concerning the expression analysis (by RNA-seq), the isolate was grown on 6 different conditions : “control” which is the same as used for the genome sequencing, *i.e* growing on AE medium for 7 days; “old”, growing the isolates on AE for 14 days instead of 7 days.; “PH5”, growing it on AE pH adjusted to pH = 5, for 7 days; “PH9”, growing it on AE, pH adjusted to pH = 9, for 7 days; “NoG”, growing it on AE without glycerol, for 7 days; “BSA”, on Barley Straw Agar medium (BSA) (40g of ground barley straw, 20 g of agar for 1000 mL of final volume), for 7 days.

DNA and RNA extraction

Mycelia from above-mentioned cultures were harvested, and finely ground mycelium was used for DNA extraction and RNA extraction.

For the DNA extraction, 1400µl of preheated (60°C) CTAB extraction buffer (100 mM Tris–HCl, pH 8.0; 20 mM EDTA, pH 8.0; 1.4M NaCl; 2% CTAB; 0.2% β-mercaptoethanol) was added to a 2 ml Eppendorf tube. The tubes were mixed thoroughly and incubated at 60°C for 1 h. The tubes were centrifuged for 5 min at 13,000 rpm. 1000 µl of the supernatant was transferred into a fresh 2 ml tube and then 1 volume of phenol/chloroform/isoamylalcohol (25:24:1) was added and the tubes were gently mixed for 5 to 10 min. Tubes were centrifuged for 20 min at 13,000 rpm and the supernatant was transferred to a new tube. RNase (20 µg/ml, DNase-free) was added and the tubes were incubated at 37°C for 30 min. One volume of phenol/chloroform/isoamylalcohol (25:24:1) was added and the tube were gently mixed for 5 to 10 min. Tubes were centrifuged for 20 min at 13,000 rpm and

the supernatant was transferred to a new tube. DNA was precipitated by adding 0.1 volume of sodium acetate (3 M, pH 5.2) and 0.6 volume of cold isopropanol. The tubes were kept at -20 °C overnight. Tubes were then centrifuged for 10 min at 13,000 rpm at 4 °C. The supernatant was discarded and the pellet was washed with 500 µl cold 70% ethanol, the pellet was spun down at 13,000 rpm for 1 min at 4 °C and the supernatant was discarded. This last step with the ethanol was repeated one more time. The precipitate was dried for 20–30 min at 37°C and then resuspended in 100 µl of sterile Tris-Buffer (10 mM Tris–HCl, pH 8.5).

Total RNA was extracted from *R. collo-cygni* mycelium from the 6 different conditions using TRIzol reagent (Invitrogen, Karlsruhe, Germany). RNA integrity was confirmed using a 2100 Bioanalyzer RNA Nanochip with the RNA 6000 Nano Kit (5067-1511) following the manufacturer's protocol (Agilent, Santa Clara, CA, USA).

Library preparation and sequencing

Whole genome sequencing of *R. collo-cygni* was performed by Eurofins Genomics GmbH, Ebersberg, Germany, using a short distance library (SD) by fragmentation and end repair of DNA (customized insert size of approx. 500 bp) on the Illumina MiSeq v3 (paired-end sequencing 2x150 bp) and a long jumping distance library (LJD) with a jumping distance of 8 kbp on the Illumina HiSeq 2000/2500 v3 (paired-end sequencing 2x300 bp).

The RNA-seq library preparation was performed using the TruSeq Stranded mRNA Library Prep Kit (RS-122-2101) (Illumina San Diego, California 92122 U.S.A.) following the manufacturer's protocol. The prepared library (Insert size: about 180 bp) was then subjected to high-throughput sequencing using the Illumina HiSeq2500 using 1 lane and multiplexed paired-end read (read 1: 101 cycles, index read: 7 cycles, read 2: 101 cycles). The sequencing was performed using the TruSeq Rapid PE Cluster Kit (PE-402-4001) and the TruSeq Rapid SBS Kits - HS (200 cycles) (FC-402-4001) (Illumina San Diego, California 92122 U.S.A.).

Genome assembly and structural annotation

The assembly was performed by ALLPATHS-LG (Gnerre, et al. 2011) using around 100 fold SD and 30 fold LJD genome covering. Gene models were generated by three de-novo prediction programs: 1) Fgenesh (Salamov and Solovyev 2000) with different matrices (trained on *Aspergillus nidulans*, *Neurospora crassa* and a mixed matrix based on different species); 2) GeneMark-ES (Ter-Hovhannisyan, et al. 2008) and 3) AUGUSTUS (Stanke, et al. 2006) with RNA-seq based transcripts as training sets. Annotation was aided by Exonerate (Slater and Birney 2005) hits of protein sequences from *Botrytis*, *Sclerotinia* and *Rhynchosporium* models to uncover gene annotation gaps. Transcripts were assembled on the RNA-seq data sets using Trinity (Grabherr, et al. 2011). The different gene models (Exonerate-mapped transcripts and Bowtie-mapped RNA-seq reads) were visualized in Gbrowse (Donlin 2009), allowing manual validation of coding sequences. The best fitting model per locus was selected manually and gene structures were adjusted by splitting or fusion of

gene models or redefining exon-intron boundaries if necessary. tRNAs where predicted using tRNAscan-SE (Lowe and Eddy 1997). Very few models with no reads were left with an automated model: only those where the model was the same for all the gene predictors. This still gives a high probability that the gene is real although not expressed in any of our media. The predicted protein sets were searched for highly conserved single (low) copy genes to assess the completeness of the genomic sequences and gene predictions. All of the 248 core-genes commonly present in higher eukaryotes (CEGs) could be identified by Blastp comparisons (e-value: 10^{-3} [(Parra, et al. 2009)]). Orthologous genes to all 246 single copy genes were searched for all proteomes by Blastp comparisons (eVal: 10^{-5}) against the single-copy families from all 21 species available from the FunyBASE (Marthey, et al. 2008). Additionally, we used BUSCO Version 3.0.1 in the Ubuntu virtual machine with the lineage specific profile library pezizomycotina_odb9 (3.156 BUSCO groups), downloaded from http://busco.ezlab.org. The analysis was performed in gene set (protein) assessment mode running the python script run BUSCO.py (Simao, et al. 2015). All genomes were analyzed using the PEDANT system (Walter, et al. 2008). To avoid misleading ortholog information based on similarity and bi-directional best hits, QuartetS (Yu, et al. 2011) was applied to retrieve a reliable ortholog matrix which was used for all comparative representations.

Phylogeny & divergence time estimation

Orthologous genes for 10 main housekeeping genes we selected from the proteomes of all 17 species are available from the FunyBASE (Marthey et al. 2008). Alignments and data preparation were done with the latest version of MEGA (Kumar et al. 2018). We performed a ClustalW pairwise alignment and multiple alingment with standard parameters for the combined proteins of all species (*An= Aspergillus nidulans; Bc = Botrytis cinerea; Bg = Blumeria graminis; Cg = Colletotrichum graminicola; Cb = Cercospora beticola; Ds= Dothistroma septosporum; Fg= Fusarium graminearum; Ff= Fusarium fujikuroi; Mf= Pseudocercospora fijiensis; Mo=Magnaporthe grisea; Pi= Piriformospora indica; Pr= Pyrenophora tritici-repentis; Pt= Pyrenophora teres f. teres; Rc= Ramularia collo-cygni; Sn= Parastagonospora nodorum; Sm= Sphaerulina musiva; Um = Ustalago maydis; Zt= Zymoseptoria tritici; Outgroup = Lb, Laccaria bicolor*) . Dating of the Zt – Rc split was done using the RelTime method (Tamura et al. 2012) where Lb was used as an outgroup and the data were calibrated using the Um to An split (Taylor & Berbee 2006).

Identification of repetitive DNA elements

Determination of repeat sequences or the interspersed repeat library for each genome was done using the RepeatModeler pipeline (http://www.repeatmasker.org/RepeatModeler/). This involved first identifying all interspersed repeat elements or families de-novo using RepeatScout (Price, et al. 2005) followed by comparing the de-novo set to known families. New families were comprised of 10 or more repeats and had a consensus sequence length longer than 50 bp. Low complexity and simple

sequence repeat families were determined with NSEG (Wootton and Federhen 1996) and Tandem-Repeat-Finder (Benson 1999), in RepeatScout, and then removed from the interspersed repeat library.

The RepBase database (Jurka, et al. 2005) was used to detect previously published families of transposable elements, pseudogenes and retroviruses. In order to determine the exact locations of the repetitive elements on the genome we used the RepBase library and the calculated library of interspersed repeat families as input for RepeatMasker (Smit, et al. 1996). RepeatMasker was also used to find and mask genomic regions of low complexity. Automated classification categorized the predicted repeat sequences into four main transposable element categories DNA transposon, long interspersed nuclear element (LINE), short interspersed nuclear element (SINE) and retrotransposon with long terminal repeats (LTRs). Intergenic distances and TE distances were calculated using bedtools (closest) (Quinlan & Hall 2010).

RNA-seq analysis

RNA-seq reads were mapped against our reference genome using TopHat (ver. 2.1.0) (Kim, et al. 2013). The gene expression levels were estimated as Fragments Per Kilobase of Million Fragments Mapped (FPKM) using the cufflinks.cuffdiff package (ver. 2.2.1) (Trapnell, et al. 2010). Genes were described as having expression evidence when FPKM => 1 between all growth conditions. FPKM values were log2 transformed and the transcripts with a fold change of expression between two conditions greater or equal to 4 (log2>=2) were considered significantly differentially expressed.

Prediction of secreted proteins and effectors

We used a pipeline which combines the use of several methods. All proteins with a canonical signal peptide were selected using SignalP3.1 (Bendtsen et al. 2004) and SignalP4.0 (Petersen, et al. 2011) (default cut offs, s-score ≥ 0.5). In addition, secreted proteins candidates with non canonical secretory motifs were selected by SecretomeP1.0 (cutoff score = 0.8) (Bendtsen, et al. 2004). This yields a combined set of proteins with both canonical and non canonical secretory leader motifs (set 1). Proteins with mitochondrial and chloroplast target signals were predicted using TargetP1.1 (RC-score < 4) (Emanuelsson, et al. 2000) and the presence of trans membrane domains using TMHMM2.0 (Krogh, et al. 2001). All these proteins were removed from set 1. The results are high confidence putatively sereted proteins. For the prediction of effectors (Table S2), the set of putative secreted proteins was submitted to EffectorP 2.0 (Sperschneider et al. 2018).

Calculation of dN/dS

Orthologous genes to all single copy genes were identified in the Zt proteome (BLASTP e-value: 10^{-10}), only (Table S5) reciprocal matches were used and globally aligned using t-coffee (default

parameters). Amino acids were replaced by codons from the cds sequence using pal2nal (Suyama et al. 2006). dN/dS were calculated with PAML(Yn00 command) (Yang & Nielsen 2000).

Statistical analyses and data visualisation

All statistical analyses were done with R (R Core Team 2015). Kruskall Wallis tests and Dunn's multiple comparisons were done using the package FSA (Ogle 2018). Boxplots and Violin plots were drawn using ggplot2 (Wickham 2009). The densitiy plot (figure 3A) was plot using the package hexbin (Carr et al. 2018) and was inspired by the methods used in (Frantzeskakis et al. 2018).

Genome alignments between the assembly of Urug2 and that of DK05 were inferred using nucmer (with --maxmatch, otherwise default options) from the MUMmer package (version 3.23) and alignments that span more than 1 kb and are more than 90% identical in sequence were plotted using Gnuplot.

References

Bendtsen JD, Jensen LJ, Blom N, Von Heijne G, Brunak S 2004. Feature-based prediction of non-classical and leaderless protein secretion. Protein Eng Des Sel 17: 349-356. doi: 10.1093/protein/gzh037

Bendtsen JD, Nielsen H, von Heijne G, Brunak S ren, Bendtsen JD. 2004. Improved Prediction of Signal Peptides: SignalP 3.0. J. Mol. Biol. 340:783–795.

Benson G 1999. Tandem repeats finder: a program to analyze DNA sequences. Nucleic Acids Res 27: 573-580.

Carr D, Lewin-Koh ported by N, Maechler M, Sarkar contains copies of lattice functions written by D. 2018. *hexbin: Hexagonal Binning Routines*. https://CRAN.R-project.org/package=hexbin.

Donlin MJ 2009. Using the generic genome browser (GBrowse). Current Protocols in Bioinformatics: 9.9. 1-9.9. 25.

Emanuelsson O, Nielsen H, Brunak S, Von Heijne G 2000. Predicting subcellular localization of proteins based on their N-terminal amino acid sequence. Journal of molecular biology 300: 1005-1016

Frantzeskakis L et al. 2018. Signatures of host specialization and a recent transposable element burst in the dynamic one-speed genome of the fungal barley powdery mildew pathogen. BMC Genomics. 19:381. doi: 10.1186/s12864-018-4750-6.

Gnerre S, et al. 2011. High-quality draft assemblies of mammalian genomes from massively parallel sequence data. Proc Natl Acad Sci U S A 108: 1513-1518. doi: 10.1073/pnas.1017351108

Grabherr MG, et al. 2011. Full-length transcriptome assembly from RNA-Seq data without a reference genome. Nature biotechnology 29: 644-652.

Jurka J, et al. 2005. Repbase Update, a database of eukaryotic repetitive elements. Cytogenet Genome Res 110: 462-467. doi: 10.1159/000084979

Kim D, et al. 2013. TopHat2: accurate alignment of transcriptomes in the presence of insertions, deletions and gene fusions. Genome biology 14: R36. doi: 10.1186/gb-2013-14-4-r36

Krogh A, Larsson B, Von Heijne G, Sonnhammer EL 2001. Predicting transmembrane protein topology with a hidden Markov model: application to complete genomes. Journal of molecular biology 305: 567-580.

Kumar S, Stecher G, Li M, Knyaz C, Tamura K. 2018. MEGA X: Molecular Evolutionary Genetics Analysis across Computing Platforms. Mol. Biol. Evol. 35:1547–1549. doi: 10.1093/molbev/msy096.

Lowe TM, Eddy SR 1997. tRNAscan-SE: a program for improved detection of transfer RNA genes in genomic sequence. Nucleic acids research 25: 955-964.

Marthey S, et al. 2008. FUNYBASE: a FUNgal phYlogenomic dataBASE. BMC bioinformatics 9: 456.

Ogle DH. 2018. *FSA: Fisheries Stock Analysis*.

Parra G, Bradnam K, Ning Z, Keane T, Korf I 2009. Assessing the gene space in draft genomes. Nucleic Acids Res 37: 289-297. doi: 10.1093/nar/gkn916

Petersen TN, Brunak S, von Heijne G, Nielsen H 2011. SignalP 4.0: discriminating signal peptides from transmembrane regions. Nature methods 8: 785-786.

Price AL, Jones NC, Pevzner PA 2005. De novo identification of repeat families in large genomes. Bioinformatics 21 Suppl 1: i351-358. doi: 10.1093/bioinformatics/bti1018

Quinlan AR, Hall IM. 2010. BEDTools: a flexible suite of utilities for comparing genomic features. Bioinformatics. 26:841–842. doi: 10.1093/bioinformatics/btq033.

R Core Team. 2015. *R: A Language and Environment for Statistical Computing*. R Foundation for Statistical Computing: Vienna, Austria https://www.R-project.org/.

Salamov AA, Solovyev VV 2000. Ab initio gene finding in Drosophila genomic DNA. Genome research 10: 516-522.

Simao FA, Waterhouse RM, Ioannidis P, Kriventseva EV, Zdobnov EM 2015. BUSCO: assessing genome assembly and annotation completeness with single-copy orthologs. Bioinformatics 31: 3210-3212. doi: 10.1093/bioinformatics/btv351

Slater GSC, Birney E 2005. Automated generation of heuristics for biological sequence comparison. BMC bioinformatics 6: 31.

Smit A, Hubley R, Green P 1996. RepeatMasker Open-3.0 http://www.repeatmasker. org.

Sperschneider J, Dodds PN, Gardiner DM, Singh KB, Taylor JM. 2018. Improved prediction of fungal effector proteins from secretomes with EffectorP 2.0. Mol. Plant Pathol. 19:2094–2110. doi: 10.1111/mpp.12682.

Stanke M, et al. 2006. AUGUSTUS: ab initio prediction of alternative transcripts. Nucleic acids research 34: W435-W439.

Suyama M, Torrents D, Bork P. 2006. PAL2NAL: robust conversion of protein sequence alignments into the corresponding codon alignments. Nucleic Acids Res. 34:W609–W612. doi: 10.1093/nar/gkl315.

Tamura T, Akutsu T 2007. Subcellular location prediction of proteins using support vector machines with alignment of block sequences utilizing amino acid composition. BMC bioinformatics 8: 466.

Taylor JW, Berbee ML. 2006. Dating divergences in the Fungal Tree of Life: review and new analyses. Mycologia. 98:838–849. doi: 10.1080/15572536.2006.11832614.

Wickham H. 2009. *ggplot2: Elegant Graphics for Data Analysis*. Springer-Verlag New York http://ggplot2.org.

Ter-Hovhannisyan V, Lomsadze A, Chernoff YO, Borodovsky M 2008. Gene prediction in novel fungal genomes using an ab initio algorithm with unsupervised training. Genome research 18: 1979-1990.

Trapnell C, et al. 2010. Transcript assembly and quantification by RNA-Seq reveals unannotated transcripts and isoform switching during cell differentiation. Nature biotechnology 28: 511. doi: 10.1038/nbt.1621

Walter MC, et al. 2008. PEDANT covers all complete RefSeq genomes. Nucleic acids research 37: D408-D411.

Wootton JC, Federhen S 1996. Analysis of compositionally biased regions in sequence databases. Methods Enzymol 266: 554-571.

Yang Z, Nielsen R. 2000. Estimating synonymous and nonsynonymous substitution rates under realistic evolutionary models. Mol Biol Evol. 17:32–43.

Yu C, Zavaljevski N, Desai V, Reifman J 2011. QuartetS: a fast and accurate algorithm for large-scale orthology detection. Nucleic Acids Res 39: e88. doi: 10.1093/nar/gkr308
